# Leptin Increases TNF-α Expression and Production through Phospholipase D1 in Raw 264.7 Cells

**DOI:** 10.1371/journal.pone.0102373

**Published:** 2014-07-21

**Authors:** Se-Min Lee, Hye-Jin Choi, Cheong-Hae Oh, Jae-Won Oh, Joong-Soo Han

**Affiliations:** 1 Biomedical Research Institute and Department of Biochemistry and Molecular Biology, College of Medicine, Hanyang University, Seoul, Korea; 2 Department of Pediatrics, College of Medicine, Hanyang University, Seoul, Korea; Fundação Oswaldo Cruz, Brazil

## Abstract

Epidemiological evidence suggests that obesity is associated with inflammation of the respiratory tract and the pathogenesis of asthma. The purpose of this study was to examine the role of phospholipase D1 (PLD1) in leptin-induced expression of the proinflammatory cytokine, tumor necrosis factor (TNF)-α, and to suggest a molecular link between obesity and respiratory tract inflammation. We investigated whether leptin, a typical adipocytokine, plays a role in the expression of TNF-α through increased PLD1 activity in Raw 264.7. Leptin enhanced the activity of PLD1 through activation of PLCγ and Src, while PLD1 siRNA decreased the leptin-induced expression and production of TNF-α. Leptin-induced PLD activation was also inhibited by a PLCγ inhibitor (PAO) and Src kinase inhibitor (PP2), indicating that PLCγ and Src kinase are upstream activators of PLD1. Down-regulation of PLD1 also completely blocked activation of p70S6K, an activator of JNK. Leptin-induced expression of TNF-α was also prevented by inhibition of p70S6K and JNK. Taken together, these results indicate that PLD1 acts as an important regulator of leptin-induced expression of TNF-α by participating in the PLCγ/Src/PLD1/PA/p70S6K/JNK pathway.

## Introduction

Leptin, a hormone/cytokine secreted by adipocytes, is an important regulator of metabolic, reproductive, and immune functions [1], [2]. Increased levels of leptin may play a role in energy homeostasis [3], neuroendocrine function [4], angiogenesis [5], and production of inflammatory mediators [6], [7]. Indeed, leptin can activate dendritic cells (DC), monocytes, and macrophages and stimulate them to express and release Th1-type cytokines [8]. Leptin also regulates pro-inflammatory immune responses in the white adipose tissue of obese mice, rats [9], and humans [10]. Despite the evidence for a role of leptin in the immune response, the intracellular signaling mechanisms involved, including those affecting of cytokine secretion, are not well understood.

Phospholipase D (PLD) catalyzes the hydrolysis of phosphatidylcholine to phosphatidic acid (PA) and choline [11]. PA, one of the enzymatic products of PLD, can be metabolically converted to diacylglycerol (DAG) and lyso-PA (LPA), both of which have second messenger roles that could contribute to the effects of PLD. Two mammalian PLD isoforms, PLD1 and PLD2, have been cloned and found to share 55% sequence homology. PLD is responsible for signaling in a variety of cellular processes, including cell proliferation [12], differentiation, cell survival, apoptosis [13], and the immune response [14]. Phospholipase C (PLC) also plays an important regulatory role in cellular immune responses [15], [16]. Activated PLCγ hydrolyses phosphatidylinositol 4,5-bisphosphate (PIP2) to DAG and inositol 1,4,5-trisphosphate (IP3), resulting in activation of protein kinase C (PKC) [17]. Several studies have shown that PLCγ is responsible for the activity of PLD in intracellular signaling events [18], [19].

mTOR is a serine/threonine protein kinase thought to be involved in inflammatory and thrombotic processes as a regulator of signal-dependent translation in various cell types [20], [21]. The best-known downstream effectors of mTOR include the ribosomal subunit S6 kinase (S6K) and the eukaryotic translation initiation factor 4E binding protein 1 (4EBP1), two regulators of mitogen-stimulated translation initiation [22]. A recent study demonstrated a critical relationship between PLD and PA in the mTOR pathway [23]. Moreover, mTOR is involved in leptin-induced inflammatory cytokine production in macrophages [24]. Leptin can induce c-jun N-terminal protein kinase (JNK) through PLC and, subsequently, PKC activation [25]. Additionally, in Kupffer cells, leptin stimulates TNF-α production via the JNK and p38 MAPK pathways [26].

In the present study, we investigated the relationship between leptin-induced TNF-α production and PLD signaling, and demonstrated that PLD1 is activated by leptin via PLCγ/Src kinase and is involved in activation of the p70S6K/JNK pathway that leads to increased TNF-α expression/production in Raw 264.7 cells.

## Materials and Methods

### Cell culture

Raw 264.7 murine macrophage cells were purchased from the American Type Culture Collection (ATCC) and cultured in Dulbecco's Modified Eagle's Medium (DMEM, low glucose) with 10% fetal bovine serum, 100 units/mL penicillin and 100 ng/mL streptomycin (Invitrogen) at 37°C with 5% CO2 in humidified air.

### Reagents

Fetal bovine serum, penicillin/streptomycin, and DMEM were purchased from Invitrogen. PAO, rapamycin, and SP600125 were obtained from Calbiochem, and [3H]-palmitic acid was from Perkin Elmer Life Sciences. 1-palmitoyl-2-arachidonoyl-sn-glycerol-3-phosphate (PA) dissolved in chloroform was purchased from Avanti Polar Lipids (Alabaster, AL, USA). The following polyclonal antibodies from Cell Signaling (Beverly, MA, USA) were used: PLD1, PLCγ, p-PLCγ, Src kinase, p-Src kinase, mTOR, p-mTOR, p-p70S6K antibody, and p70S6K. GAPDH and PLCγ were from Santa Cruz Biotechnology (Santa Cruz, CA, USA).

### Semi-quantitative and real-time PCR

Total cellular RNA was isolated using TRizol reagent (Invitrogen) according to the manufacturer's instructions. cDNA synthesis was performed using Superscript kit (Invitrogen). Each PCR procedure was carried out in optimal MgCl2 concentration, annealing temperature, and cycle number for the linear amplification range. PCR products were analyzed by 1% agarose gel electrophoresis. Real-time PCR was performed on a CFX96 Real time system using iQ SYBR green supermix (Bio-Rad). All gene expression values were normalized to those of GAPDH. The primer sequences were as follows: TNF-α sense (5′AGCCCACG-TCGTAGCAAACCACCAA3′) and antisense (5′AACACCCATTCCC-TTC-ACAGAGCAAT3′) (PCR product, 200 bp); PLD1 sense (5′ACTCTGTCCAAAGTTAACA TGT CACTG3′) and antisense (5′GGCTTTGTCTTGAGCAGCTCTCT3′) (PCR product, 245 bp); GAPDH sense (5′CATGAGAAGTATGACAACAGCCT3′) and antisense (5′AGTCCTTCCAC GATA-CCAAAGT3′) (PCR product, 300 bp).

### Transient transfection with plasmid DNA in Raw 264.7 cells

Raw 264.7 cells were transiently transfected using 5 µg each of pEGFP-C1 (vector), EGFP-PLD1, EGFP-dominant negative PLD1, EGFP-PLD2, or EGFP-dominant negative PLD2 plasmid using Lipofectamine reagents (Invitrogen). 48 h after transfection the cells were serum-starved for 18 h and then treated with leptin.

### Western blot analysis

Cells were first lysed in 20 mM Tris, pH 7.5, containing 150 mM NaCl, 1 mM EDTA, 1 mM EGTA, 2.5 mM sodium pyrophosphate, 1% Triton X-100, 1 mM phenylmethylsulfonyl fluoride, and 1 mM Na_3_VO_4_. Protein samples of 10 to 20 µg were loaded onto SDS-polyacrylamide gels (10%), electrophoresed, and transferred to nitrocellulose membranes (Amersham Biosciences). After being blocked with 5% dried skim milk for 2 h, the membranes were incubated with primary antibodies, followed by horseradish peroxidase-conjugated secondary antibody (1∶2000, New England Biolabs, Beverly, MA), and specific bands were detected by ECL (Amersham Biosciences).

### Determination of PLD activity

PLD activity was determined by the formation of phosphatidylbutanol (PBt), the product of PLD-mediated transphosphatidylation in the presence of butanol as described previously, with a slight modification [27]. The cells, which had been incubated for the indicated times in 6-well plates, were radioactively labeled with 2 µCi/mL of [^3^H]-palmitic acid in serum-free medium for 20 h, then pretreated with 0.3% (v/v) 1-butanol for 15 min before stimulation with leptin. Additionally, cells were preincubated with PAO or PP2 for 30 min after being labeled with [^3^H]-palmitic acid, and serum-starved for 18 h. After treatment with leptin for 30 min, they were quickly washed with ice-cold phosphate-buffered saline and suspended in ice-cold methanol. Lipids were extracted according to the Bligh and Dyer method [28], and PBt was separated by TLC using the acetate/isooctane/acetic acid/water (110∶50∶20∶100, v/v) solvent system. Regions corresponding to authentic PBt bands were identified with 0.002% (w/v) primulin in 80% (v/v) acetone, scraped, and counted using a liquid scintillation counter.

### ELISA

Cell supernatants were collected after leptin treatment and the concentration of TNF-α was measured with commercial ELISA kits (Bender Medsystems, Vienna, Austria) according to the manufacturer's instructions. The concentration of each sample was determined from a standard curve, and ranged from 3.13 to 2000 pg/mL. The means of triplicate ELISA values for each of the doses of PLD1 knockdown and TNF-α protein expression were calculated by linear regression.

### Statistical analysis

All experiments were repeated at least 3 times. Statistical comparisons were made using one-way Student's t-test or multi-factorial ANOVA. GraphPad Prism (version 6; GraphPad Software, San Diego, Calif., USA) was used for statistical analysis. Values of p<0.05 were considered statistically significant.

## Results

### PLD1 regulates leptin-induced TNF-α expression and production in Raw 264.7 cells

**Figure 1:**
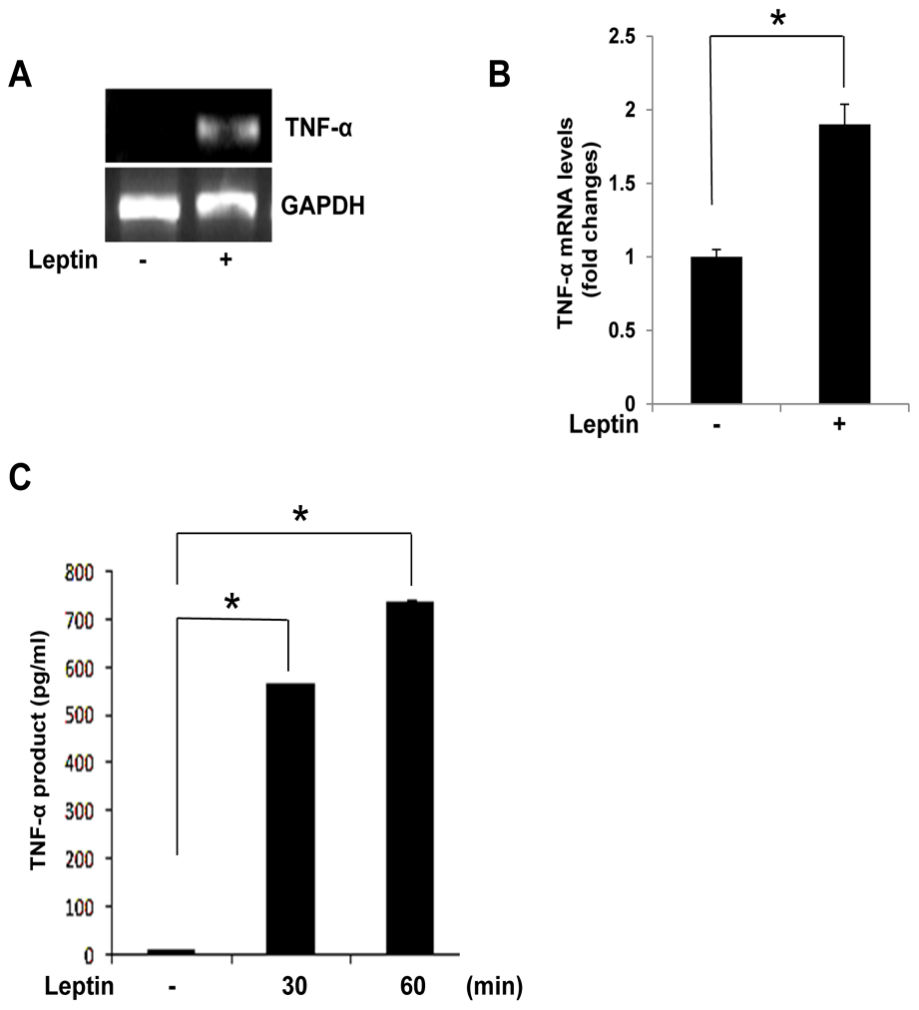
Effect of leptin on TNF-α expression in Raw 264.7 cells. (A,B) Raw 264.7 cells were treated with leptin (20 nM) for 30 min. Total RNA was isolated using TRIzol reagent, and mRNA levels were determined by semi-quantitative and real-time RT-PCR with primers for TNF-α or GAPDH. *p<0.05 vs control. (C) For ELISA, cells in 96-well culture plates were treated with leptin (20 nM) for the indicated times. Results are the mean ± S.E. for each group of samples. Data are means ± S.E. of eight values. *p<0.05 vs control.

**Figure 2:**
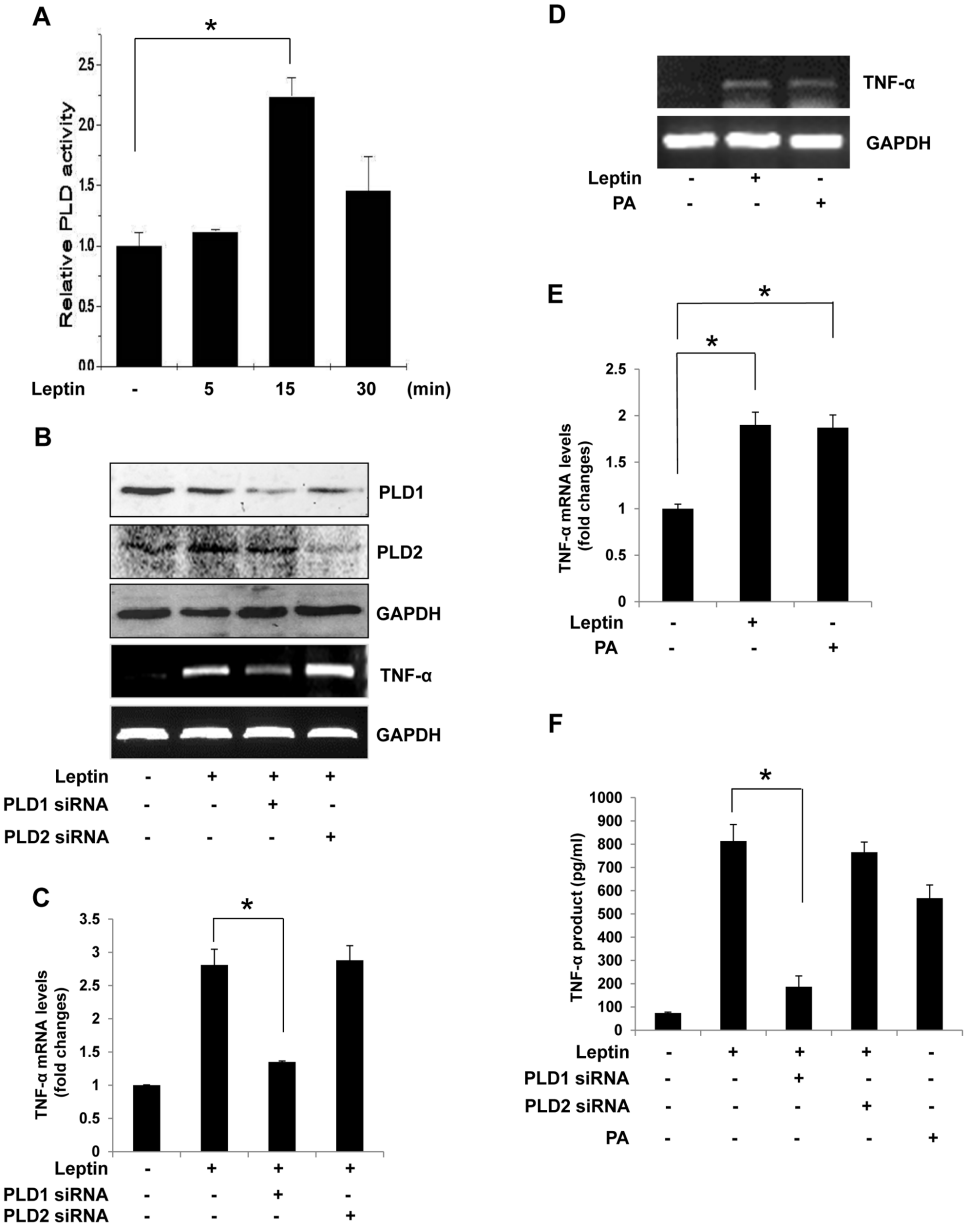
Effect of PLD knockdown on leptin-induced TNF-α expression in Raw 264.7 cells. (A) Raw264.7 cells were labeled with 2 µCi/mL [^3^H]-palmitic acid and stimulated with leptin (20 nM) for the indicated times. PLD activity was determined by estimating the amount of [^3^H]-PBt in the presence of 1-butanol. Results are the mean ± S.D. of three independent experiments. *p<0.05 vs control. (B,C) Raw264.7 cells were transiently transfected with 200 nM PLD1 siRNA or PLD2 siRNA for 48 h and were stimulated with leptin (20 nM) for 30 min. Cells lysates were subjected to Western blotting. The cells were harvested and total RNA was isolated using TRIzol reagent, and mRNA levels were determined by semi-quantitative and real-time RT-PCR with primers for TNF-α or GAPDH. *p<0.05 vs leptin-treated control. (D,E) Cells were treated with leptin (20 nM) or PA (10 µM) for 30 min and analyzed. mRNA levels were determined by semi-quantitative and real-time RT-PCR with primers for TNF-α or GAPDH. *p<0.05 vs control. (F) Cells on 96-well culture plates were transfected with 200 nM PLD1 siRNA or PLD2 siRNA for 48 h and then stimulated with leptin (20 nM) for 1 h. Cells were also treated with PA (10 µM) for 1 h. Results are the mean ± S.E. amounts of TNF-α measured by ELISA for each group of samples. Data are means ± S.E. of eight values. *p<0.05 vs leptin-treated control.

To determine whether leptin induces production of TNF-α, Raw 264.7 cells were treated with leptin for the indicated times. Treatment of the cells with leptin increased TNF-α expression (Figure 1A,B) and production(Figure 1C)). To determine the role of PLD in this increase, we measured the activity of PLD. As shown in Figure 2A, leptin increased PLD activity in treated cells nearly 2.2-fold within 15 min. Next, we investigated which isozymes of PLD in Raw 264.7 cells were involved in the observed effect. We found that knockdown of PLD1 by siRNA significantly reduced the expression and production of TNF-α, while PLD2 siRNA did not. This suggested that only PLD1 had an impact on the expression (Figure 2B,C) and production of TNF-α (Figure 2F). A similar effect on TNF-α was also induced by PA, the end product of PLD (Figure 2D,E), confirming that PLD1 plays an important role in the expression and production of TNF-α in response to leptin.

### Leptin activates PLCγ/Src kinase, resulting in increased PLD activity and TNF-α expression and production

**Figure 3:**
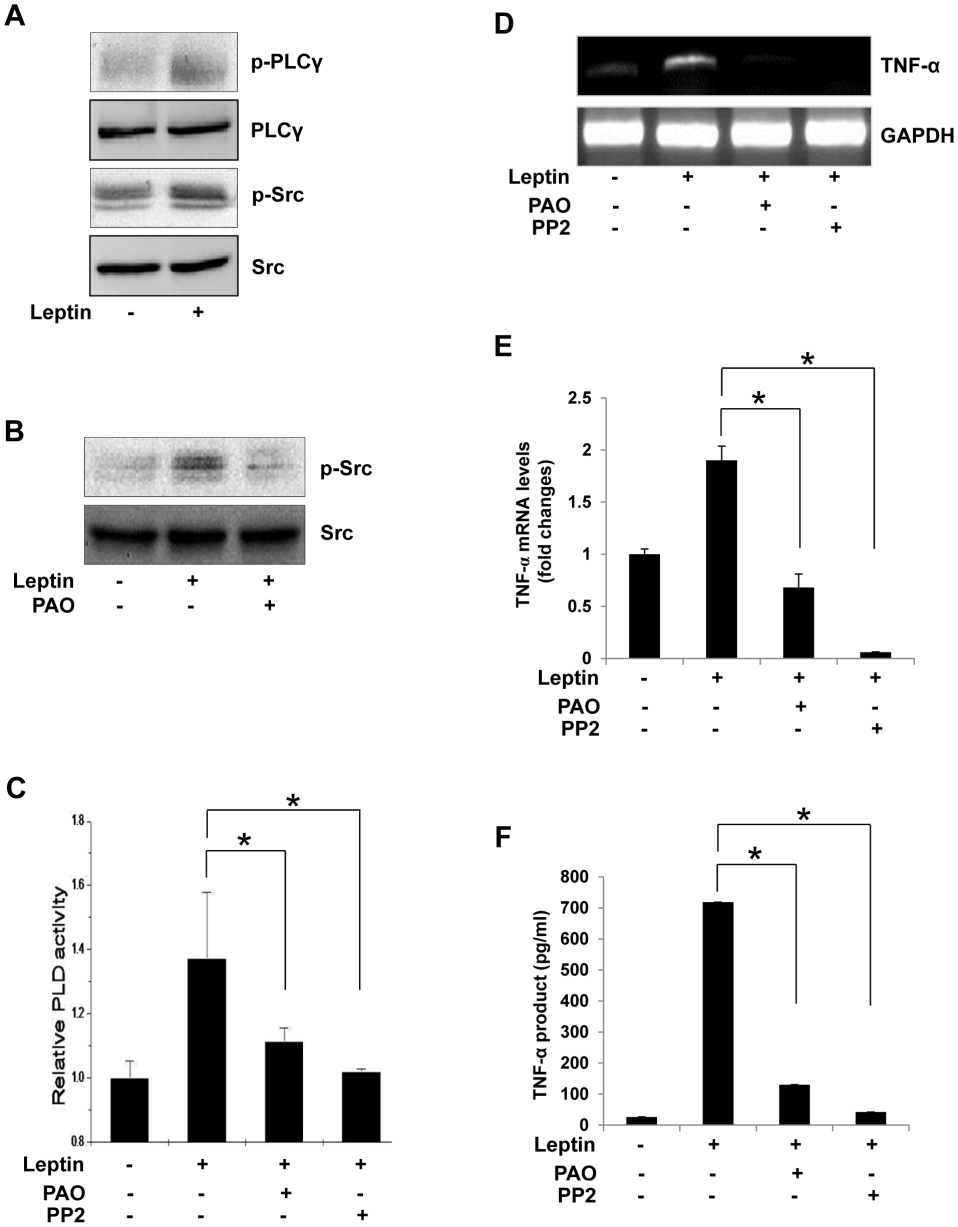
Effect of the PLCγ inhibitor (PAO) and Src kinase inhibitor (PP2) on leptin-induced TNF-α expression in Raw 264.7 cells. (A) Raw 264.7 cells were treated with leptin (20 nM) for 5 min. Cells were harvested, and cell extracts were subjected to immunoblotting for PLCγ and Src kinase, respectively. (B) Cells were pretreated with PAO (20 µM) for 30 min and then stimulated with leptin (20 nM) for 5 min. The cell lysates were then analyzed by Western blotting using total Src and p-Src antibodies. (C) Cells were labeled with 2 µCi/ml [^3^H]-palmitic acid and then pretreated with PAO or PP2 (20 µM) for 30 min before stimulation with leptin (20 nM) for 15 min. PLD activity was determined by estimating the formation of [^3^H]-PBt in the presence of 1-butanol. Results are the mean ± S.D. of three independent experiments. *p<0.05 vs leptin-treated control (D,E) Cells were pretreated with PAO or PP2 (20 µM) for 30 min and then stimulated with leptin (20 nM) for 30 min. Total RNA was isolated using TRIzol reagent, and mRNA levels were determined by semi-quantitative and real-time RT-PCR with primers for TNF-α or GAPDH. *p<0.05 vs leptin-treated control. (F) Cells in 96-well culture plates were pretreated with PAO or PP2 (20 µM) for 30 min and then stimulated with leptin (20 nM) for 1 h. Results are the mean ± S.E. amounts of TNF-α measured by ELISA for each group of samples. Data are means ± S.E. of eight values. *p<0.05 vs leptin-treated control.

To investigate the mechanism by which leptin activates PLD1, we examined PLCγ signaling, as PLCγ activation was previously shown to increase PLD activity [18]. As shown in Figure 3A, leptin induced the phosphorylation of both PLCγ and Src kinase. Therefore, we asked whether phosphorylation of Src was dependent on the presence of PLCγ. PAO, a PLCγ inhibitor, completely blocked the phosphorylation of Src kinase induced by leptin (Figure 3B). Next, to determine whether PLCγ/Src kinase affected PLD activation in response to leptin, cells were pretreated with a specific PLCγ inhibitor (PAO) or a Src kinase inhibitor (PP2). As shown in Figure 3C, pretreatment with PAO or PP2 completely inhibited PLD activation. Furthermore, when the activity of PLCγ and Src kinase was blocked, significant inhibition of TNF-α expression (Figure 3D,E) and its production (Figure 3F) were observed, demonstrating that the PLCγ/Src kinase pathway is located upstream of PLD1. Taken together, these results establish that PLCγ/Src kinase activation regulates the leptin-induced PLD activation that leads to TNF-α expression in Raw 264.7 cells.

### mTOR/JNK signaling is required for leptin-induced TNF-α expression and production

**Figure 4:**
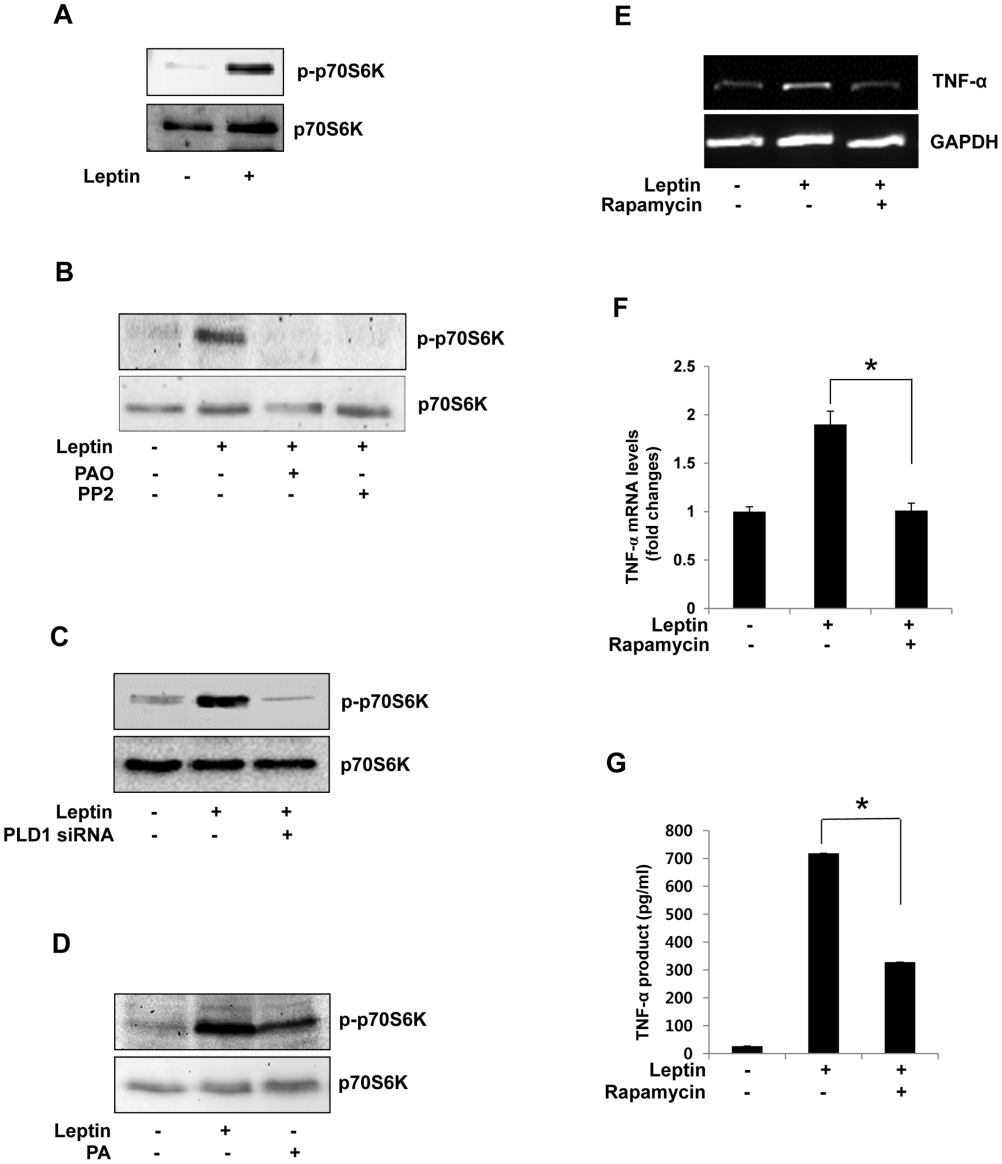
Involvement of p70S6K in leptin-induced TNF-α expression in Raw 264.7 cells. (A) Cells were treated with leptin (20 nM) for 5 min and then harvested, and the amounts of total p70S6K and p-p70S6K were determined by Western blotting. (B) Cells were pretreated with PAO or PP2 (20 µM) for 30 min and then treated with leptin as above. (C) Cells were transiently transfected with 200 nM PLD1 siRNA for 48 h and stimulated with leptin (20 nM) for 30 min. Cell lysates were analyzed as above. (D) Cells were treated with PA (10 µM) for 1 h, and cell lysates were analyzed as above. (E,F) Cells were pretreated with rapamycin (50 µM) for 30 min and then stimulated with leptin (20 nM) for 30 min. Total RNA was isolated using TRIzol reagent, and mRNA levels were determined by semi-quantitative and real-time RT-PCR with primers for TNF-α or GAPDH. *p<0.05 vs leptin-treated control (G) Cells in 96-well culture plates were pretreated with rapamycin (100 nM) for 30 min and then stimulated with leptin (20 nM) for 1 h. Results are the mean ± S.E. amounts of TNF-α measured by ELISA for each group of samples. Data are means ± S.E. of eight values. *p<0.05 vs leptin-treated control.

The mTOR pathway integrates insulin and nutrient signaling in many cells [29], [30]. We observed that leptin stimulated the phosphorylation of p70S6K (Figure 4A). To determine whether the PLCγ/Src kinase pathway was required for this effect, cells were pretreated with the specific PLCγ inhibitor (PAO) or the Src kinase inhibitor (PP2), respectively. As shown in Figure 4B, these treatments completely inhibited he p70S6K phosphorylation. We also studied the role of PLD1 in this effect using siRNA transfection; this completely abolished p70S6K phosphorylation in response to leptin (Figure 4C). To confirm the role of PLD1, we treated cells with PA and found that it also increased p70S6K phosphorylation, even in the absence of leptin (Figure 4D). As a final step, we investigated whether TNF-α expression and production were regulated by mTOR. Pretreatment of cells with rapamycin, a specific inhibitor of mTOR, completely blocked TNF-α expression (Figure 4E,F) and production in response to leptin (Figure 4G).

**Figure 5:**
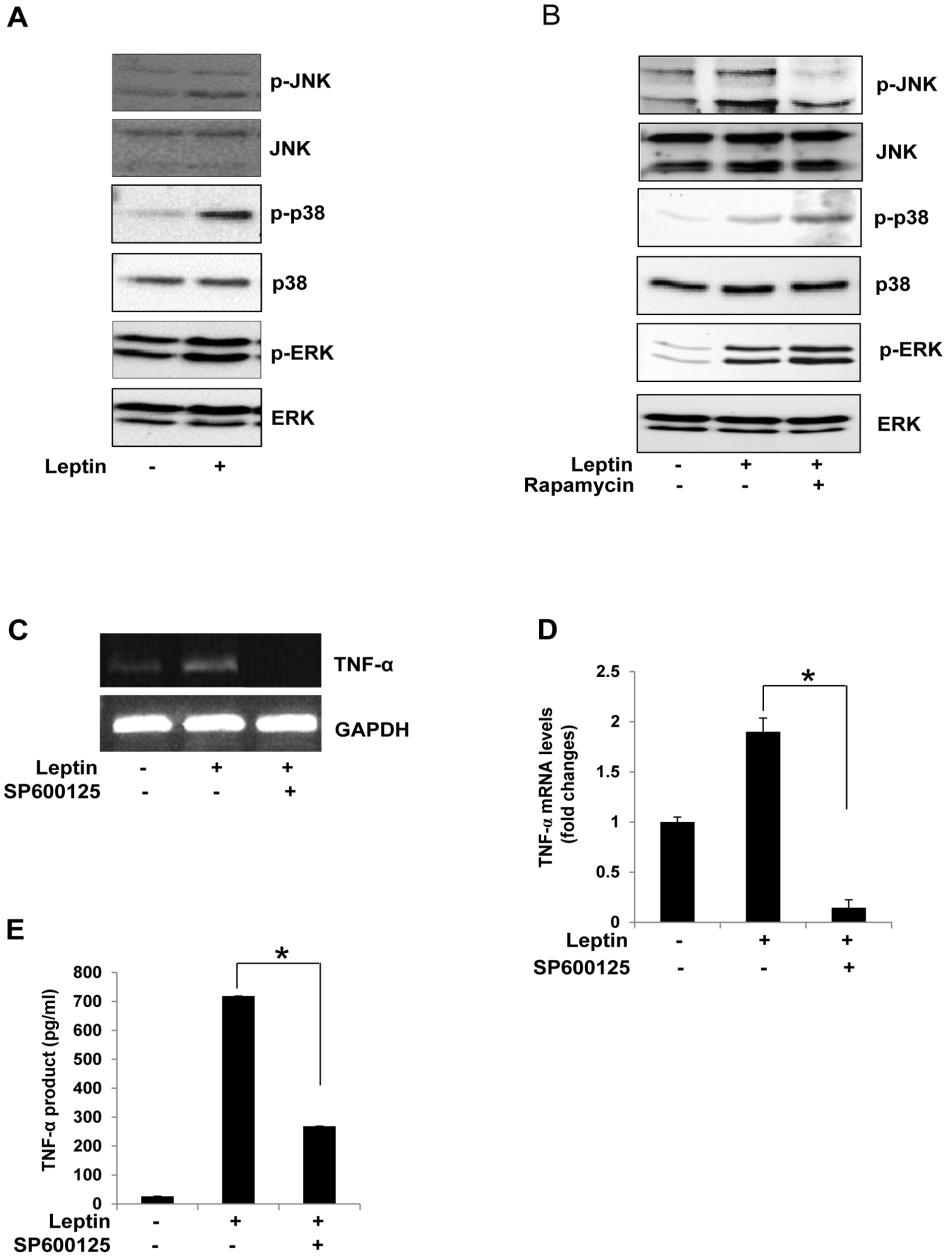
Effect of JNK phosphorylation on leptin-induced TNF-α expression in Raw 264.7 cells. (A) Raw 264.7 cells treated with leptin (20 nM) for 5 min were harvested, and cell extracts were subjected to immunoblotting for active and total MAPKs. (B) Cells were pretreated with rapamycin (100 nM) for 30 min and then stimulated with leptin (20 nM) for 5 min. Phosphorylated JNK was determined by Western blotting. (C,D) Cells were pretreated with SP600125 (50 µM) for 30 min and then stimulated with leptin (20 nM) for 30 min. Total RNA was isolated using TRIzol reagent, and mRNA levels were determined by semi-quantitative and real-time RT-PCR with primers for TNF-α or GAPDH. *p<0.05 vs leptin -treated control. (E) Cells in 96-well culture plates were pretreated with SP600125 (50 µM) for 30 min and then stimulated with leptin (20 nM) for 1 h. Results are the mean ± S.E. amounts of TNF-α measured by ELISA for each group of samples. Data are means ± S.E. of eight values. *p<0.05 vs leptin -treated control.

One of the targets of leptin is MAPK [26], And we confirmed that leptin stimulated the phosphorylation of MAPKs: treatment with leptin increased the phosphorylation of JNK, p38MAPK, and ERK (Figure 5A). Interestingly, rapamycin only blocked the phosphorylation of JNK not the other MAPKs, suggesting that only JNK signaling was mTOR-dependent (Figure 5B). Finally, we investigated the effect of JNK activation on the TNF-α expression and production in leptin-treated Raw264.7 cells. The JNK inhibitor, SP600125, significantly inhibited the expression (Figure 5C,D) and production (Figure 5E) of TNF-α, which was induced by leptin. These results demonstrate that both the expression and production of TNF-α in response to leptin are regulated by the mTOR/JNK pathway in Raw 264.7 cells.

## Discussion

The effect of leptin on cells of the immune system such as macrophages and B cells is typically achieved via stimulation of Th1 cytokine expression [31], [32]. Regulation of leptin-induced signaling pathways is a key step in immune-related diseases. Interestingly leptin potentiates the production of proinflammatory cytokines (including TNF-α and IL-6) in macrophages in response not only to LPS but also to ozone exposure [33], [34]. However, the leptin-induced intracellular signaling pathways leading to cytokine expression are not yet fully understood. Here, we investigated the molecular mechanism of PLD1-mediated TNF-α expression and production in response to leptin. Many researchers used a supraphysiological concentration of leptin to find out mechanisms for leptin-induced proinflammatory cytokines in several cell models [6], [7], [24], [35].

In the present study, leptin increased PLD1 activity, reaching a maximum value at 15 min (Figure 1), without a concurrent change in PLD1 and PLD2 expression (data not shown), which suggests that PLD1 activation was caused by leptin-regulated signaling. A PLD-knockdown experiment using PLD1 and PLD2 siRNAs showed that leptin-induced TNF-α expression and production were coupled to PLD1 activation in Raw 264.7 cells, these studies suggest a possible functional role of PLD1 in cytokine expression and production. PLD is a key enzyme that transduces direct and receptor-mediated signaling from several molecules, in particular Ras, Src kinase, and PLCγ [14], [19], [36]. PLC activation is required for the signal transduction events leading to inflammatory responses [16], [37]. In LPS-stimulated macrophages and human endothelial cells, proinflammatory cytokine secretion and expression are dependent on Src kinase [38], [39], [40]. Therefore, the role of Src kinase in immune response is rapidly emerging. Prior to this report, however, the relationship between leptin and PLCγ/Src kinase, which regulate PLD1 activation in the immune response, had not been examined. In the present study, we showed that leptin stimulated the activation of PLCγ and Src kinase. As expected, PAO and PP2 attenuated not only PLD1 activation but also TNF-α production, thus supporting the idea that the PLCγ/Src kinase pathway regulates PLD1 activation and TNF-α production induced by leptin in Raw 264.7 cells.

Accumulating evidence shows that the mTOR signaling pathway regulates inflammatory mechanisms in a number of cell types [41], [42]. Activation of mTOR has been associated with cytokine and hormone receptor signaling that involving interferon and insulin [43], [44]. Indeed, in macrophages, leptin was shown to induce time-dependent phosphorylation of 4EBP1 and p70S6K via a rapamycin-sensitive mechanism [24]. Yet, the pathway responsible for p70S6K activation in TNF-α production is not fully understood. Therefore, we hypothesized that leptin might modulate TNF-α production via the mTOR pathway. In the present study, we found that phosphorylation of p70S6K was increased by leptin, and that rapamycin could suppress TNF-α expression and production. These results demonstrate that mTOR is critically important in leptin signaling for macrophage activation leading to cytokine production.

The MAPK family plays important roles in the expression of proinflammatory cytokines in many cell types [45], [46]. Among these kinases, JNK is another candidate for regulation by PLD [47], and is involved in inflammation [48], [49]. Leptin is reported to induce JNK phosphorylation through PLC and, subsequently, PKC activation [25]. Leptin also stimulates production of TNF-α via the p38 MAPK and JNK pathways in LPS-treated cells [26]. Furthermore, it activates the MAPK pathways to mediate anti-apoptotic effects in mononuclear cells [50]. Thus the MAPK pathways appear to be essential in a wide range of immune responses. In the present study, phosphorylation of JNK was increased by leptin, and decreased when the cells were pretreated with rapamycin. This led us to think that leptin-induced JNK activation was involved in the mTOR pathway. As expected, a JNK inhibitor, SP600125, attenuated the leptin-induced expression and production of TNF-α, thus raising the possibility that JNK regulates TNF-α expression and production induced by leptin in Raw 264.7cells.

**Figure 6:**
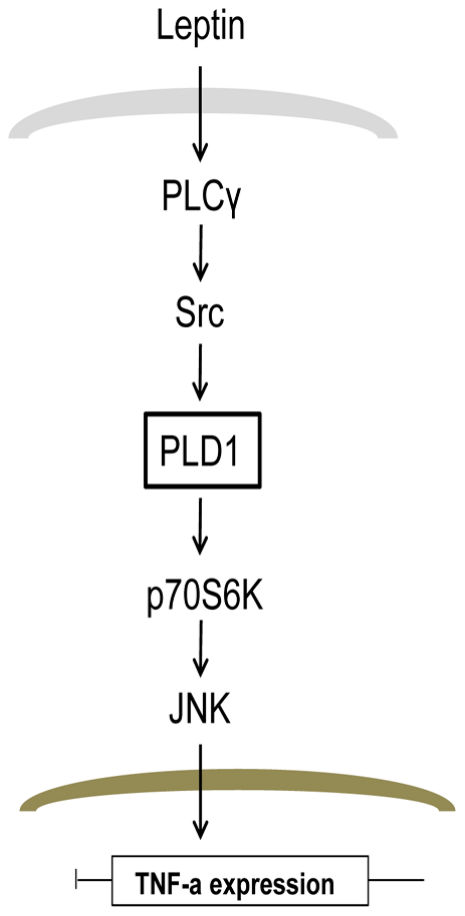
PLD1 activation is crucial for leptin-induced TNF-α expression and production in Raw264.7 cells. Our novel finding in this experiment is that leptin-induced TNF-α expression is controlled by PLD1 activation.

In conclusion, the new evidence found in our study suggests that leptin-induced PLD1 activation occurs via PLCγ/Src, and that activation of PLD1 is essential for TNF-α expression and production via the mTOR/JNK pathway in Raw 264.7 cells (Figure 6).
